# Pediatric Floating Elbow: A Case Report of Combined Supracondylar Humerus and Distal Both Bone Forearm Fractures in an 11-Year-Old Boy

**DOI:** 10.7759/cureus.103007

**Published:** 2026-02-05

**Authors:** Sk Julfikar Hossain, Basant Rai, Anurag S Sekhon

**Affiliations:** 1 Orthopedics and Traumatology, Government Medical College, Amritsar, IND; 2 Orthopedics, Government Medical College, Amritsar, IND; 3 Orthopedic Surgery, Government Medical College, Amritsar, IND

**Keywords:** compartment syndrome, distal forearm fracture, floating elbow, k-wire fixation, pediatric trauma, supracondylar humerus fracture

## Abstract

The term “floating elbow” describes a rare injury pattern in children involving ipsilateral fractures of the humerus and forearm bones. These high-energy injuries are frequently associated with neurovascular compromise and carry a risk of compartment syndrome. We report an 11-year-old boy who sustained a fall from height, resulting in a left supracondylar humerus fracture (Gartland type III) and ipsilateral distal radius and ulna fractures. Closed reduction and percutaneous K-wire fixation were performed for the supracondylar fracture, and open reduction with K-wire fixation was done for the distal radius, under general anesthesia. Postoperatively, the limb was immobilized in an above-elbow posterior splint. The neurovascular status remained intact throughout the course. The patient achieved radiological union and a full range of motion at three months. Early recognition and prompt fixation of both fractures yield favorable outcomes in pediatric floating elbow injuries. Careful monitoring for compartment syndrome remains essential.

## Introduction

The term “floating elbow” was first coined by Stanitski and Micheli in 1980 to describe a simultaneous fracture of the humerus and forearm bones in the same limb, effectively rendering the elbow "disconnected" from the rest of the extremity [1]. The incidence of such injuries in children is relatively rare, estimated between 2% and 13% of all pediatric supracondylar fractures [2]. The most common mechanism of injury is a fall on an outstretched hand from height, which transmits the force proximally, resulting in a supracondylar fracture and, in some cases, distal forearm fractures [3]. These injuries are frequently associated with soft tissue swelling, vascular compromise, and risk of compartment syndrome, making early recognition and management essential [4]. There remains debate over the ideal treatment sequence, whether to fix the humerus or the forearm first, but most authors advocate closed reduction and percutaneous (CRPP) pinning of both fractures during the same anesthesia session to reduce manipulation and minimize complications [5]. Several authors have attempted to classify pediatric floating elbow injuries. Templeton and Graham categorized them by the level of forearm involvement, whereas Rasool grouped them by fracture displacement and pattern. However, no universally accepted classification system exists for children, and most authors continue to describe them descriptively [2,6].

Earlier literature described pediatric floating elbow injuries as having a high risk of complications, particularly compartment syndrome and neurovascular compromise. However, more recent pediatric studies and systematic reviews suggest that these injuries are not as problematic as previously believed, with most children achieving good to excellent functional outcomes when managed with timely stabilization, selective fixation, and vigilant postoperative monitoring.

## Case presentation

An 11-year-old right-handed boy presented to the emergency department with complaints of severe pain, swelling, and deformity around his left elbow and wrist following a fall from a roof approximately 8 feet high. The child had fallen onto his outstretched hand. On initial examination, there was obvious deformity and diffuse swelling over the left elbow and distal forearm. The overlying skin was intact, and there were no open wounds. Tenderness was elicited over the supracondylar region of the humerus as well as over the distal forearm. The elbow and wrist movements were painful and restricted. The distal neurovascular examination revealed palpable radial and ulnar pulses with normal capillary refill, and there was no sensory or motor deficit in the median, ulnar, or radial nerve territories.

**Figure 1:**
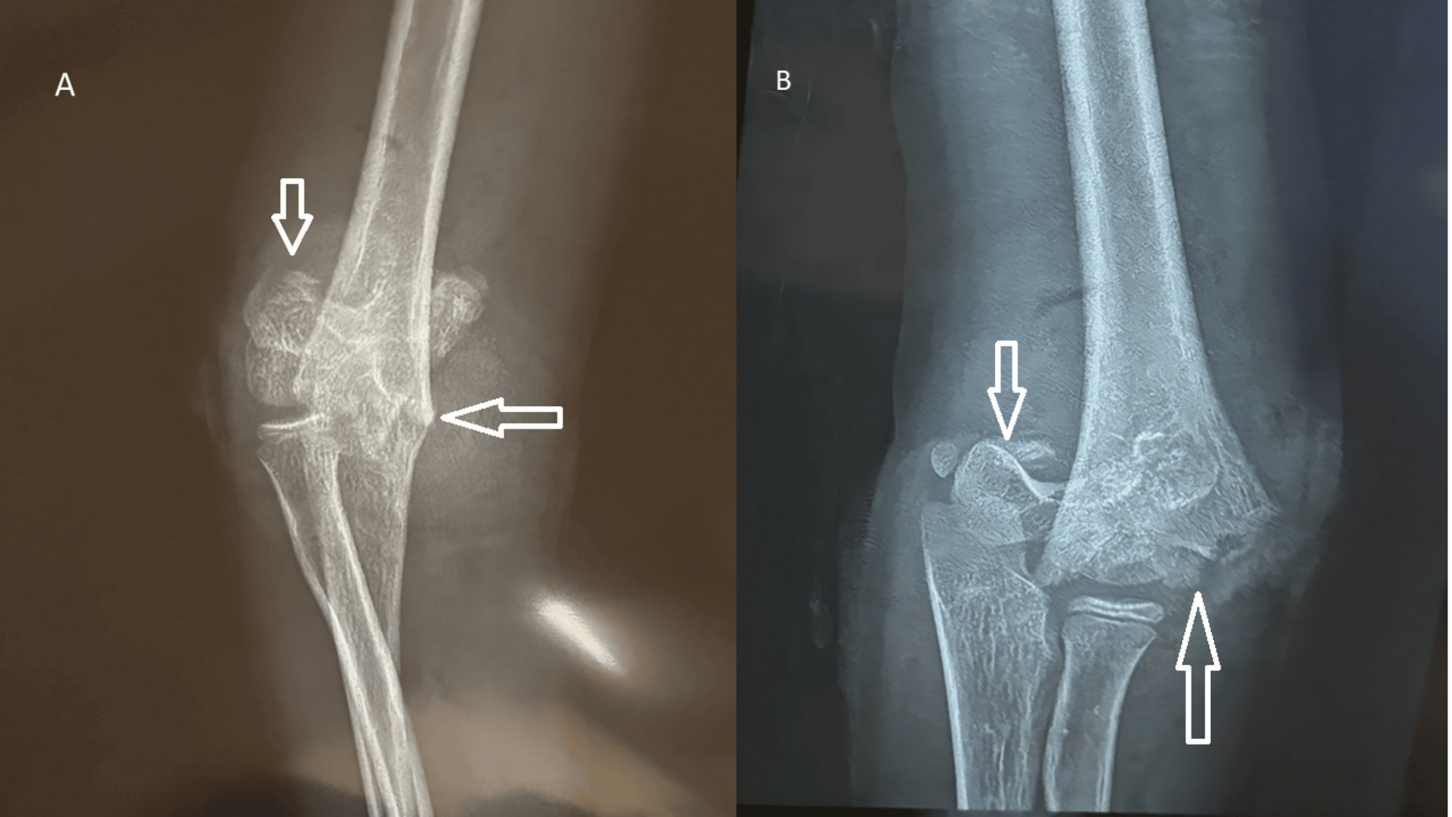
AP (A) and oblique (B) radiographs of the elbow showing a Gartland type III extension-type supracondylar fracture of the humerus A true lateral view could not be obtained due to patient discomfort; a slightly oblique view was acquired instead AP: anteroposterior

**Figure 2:**
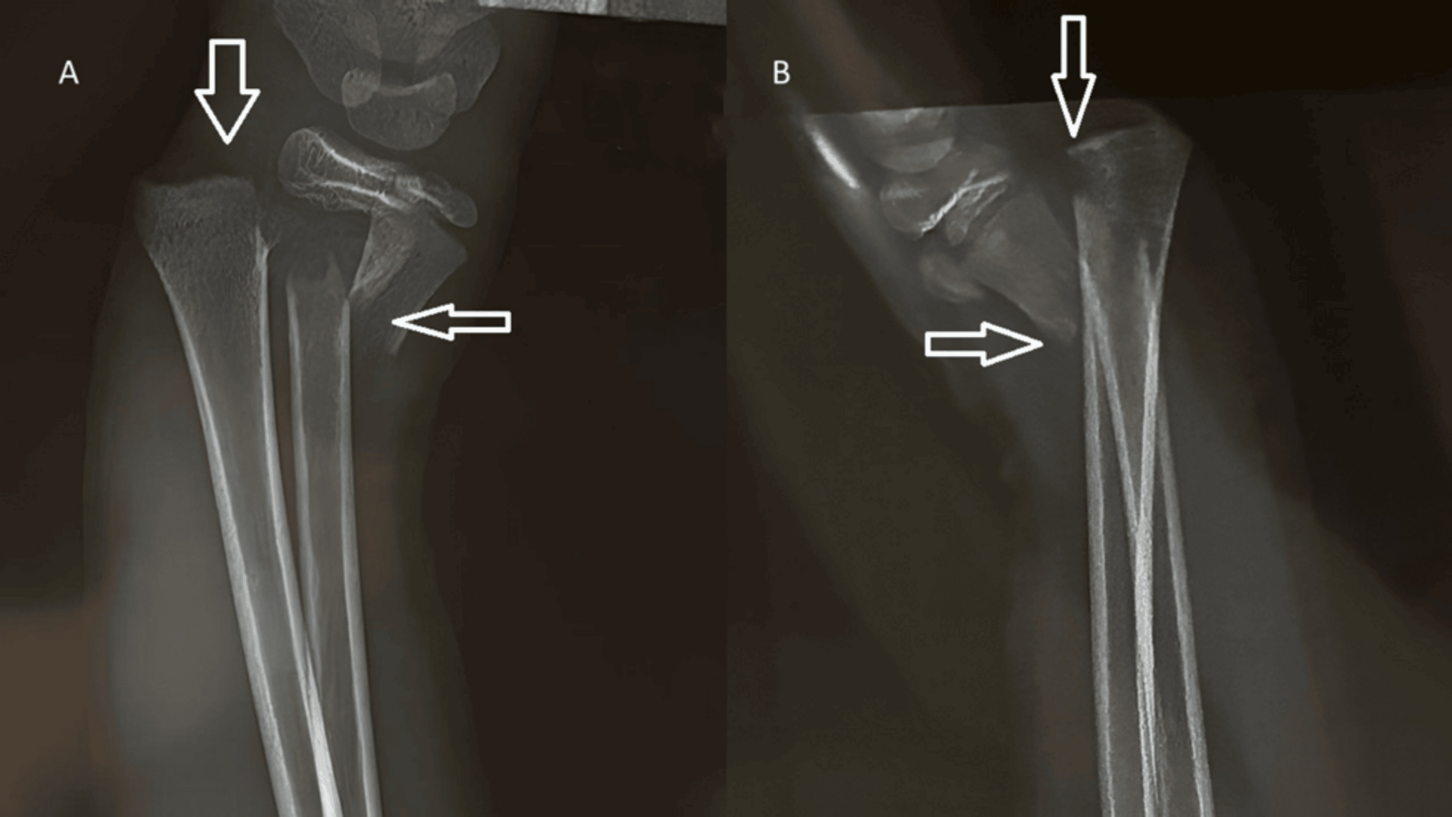
AP (A) and lateral (B) views of the left wrist showing transverse fractures of the distal radius and ulna at the metaphyseal level AP: anteroposterior

**Figure 3:**
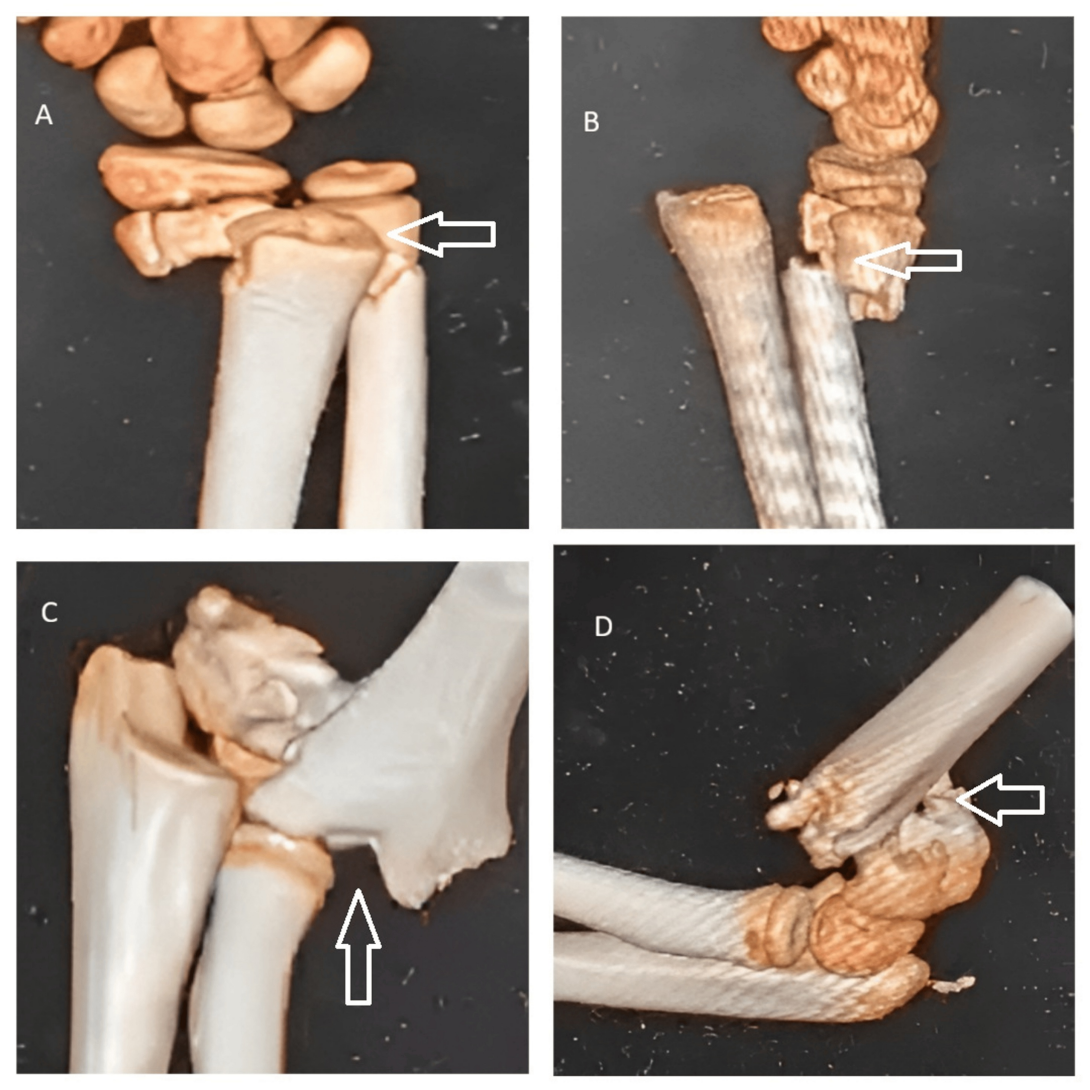
Preoperative CT images of the left elbow and wrist demonstrating fracture configuration Preoperative CT scans of the left upper limb showing metaphyseal fractures of the distal radius and ulna with minimal comminution (A,B) and a completely displaced, comminuted supracondylar humerus fracture (C,D). The images confirm extra-articular involvement and aid in surgical planning for fixation CT: computed tomography

Radiographs of the left upper limb revealed a completely displaced extension-type supracondylar fracture of the humerus, classified as Gartland type III (Figure 1), along with transverse fractures of the distal radius and ulna at the metaphyseal level (Figure 2). A CT scan of the elbow and wrist was performed because the initial radiographs did not adequately delineate the fracture geometry, and advanced imaging was required to confirm the degree of displacement, rule out physeal or intra-articular extension, and provide medicolegal documentation for operative planning. The CT confirmed a completely displaced, comminuted supracondylar fracture of the distal humerus without articular involvement, and the distal radius fracture was metaphyseal and extraphyseal, with no involvement of the physis, ruling out a Salter-Harris II injury and ulna with minimal comminution (Figure 3). The diagnosis of a pediatric “floating elbow” injury was made based on these imaging findings.

**Figure 4:**
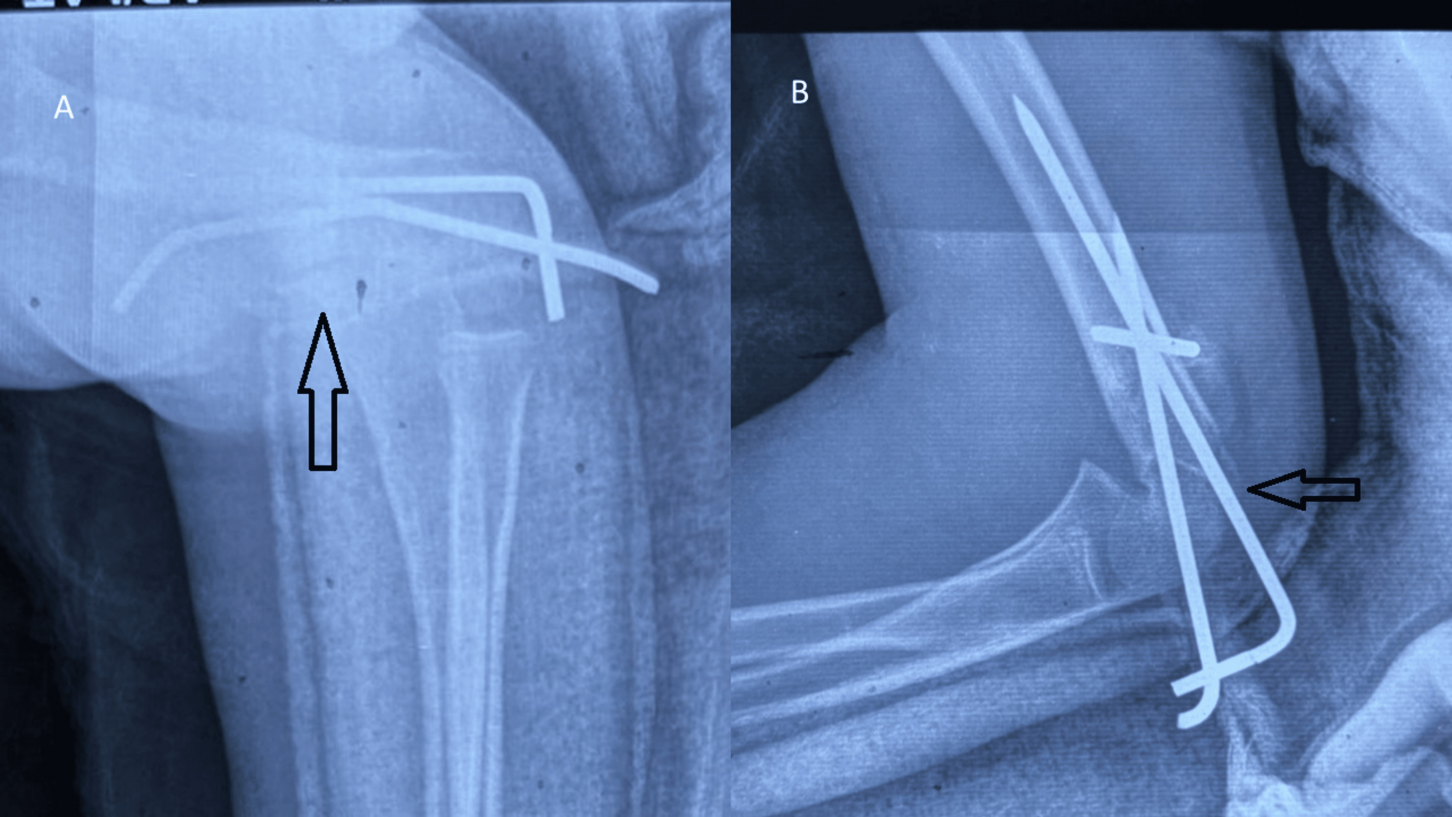
Immediate postoperative radiographs of the left elbow, showing stable fixation of the supracondylar humerus fracture (A) Anteroposterior radiograph of the left elbow showing satisfactory reduction and stable crossed Kirschner wire fixation of the supracondylar humerus fracture. (B) Lateral radiograph of the left elbow demonstrating maintained alignment and stable fixation of the supracondylar humerus fracture

**Figure 5:**
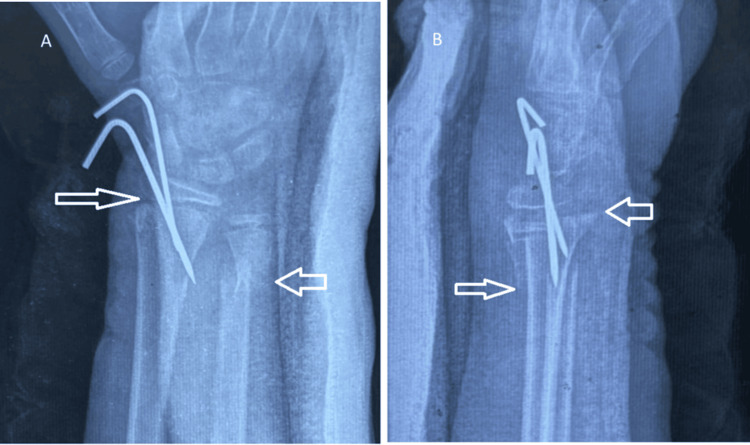
Immediate postoperative radiographs of the left wrist showing stable fixation of the distal radius fracture Immediate postoperative anteroposterior (A) and lateral (B) radiographs of the left wrist demonstrating anatomical reduction and stable fixation of the distal radius fracture with two percutaneous Kirschner wires inserted through a volar approach. The distal ulna fracture appears well-aligned and was managed conservatively

After obtaining informed consent from the parents, the patient was taken for surgery under general anesthesia. Closed reduction of the supracondylar humerus fracture was performed under fluoroscopic guidance using the gentle traction-counter-traction technique. Once satisfactory alignment was achieved, the fracture was stabilized with three 1.6-mm Kirschner wires. The distal radius fracture, however, could not be adequately reduced by closed manipulation. Therefore, an open reduction was performed through a volar (Henry) approach, exposing the fracture site. After anatomical reduction was achieved, stabilization was accomplished with two 1.2-mm K-wires. The ulna fracture was managed nonoperatively because additional fixation would have required further soft-tissue dissection, which could increase compartmental pressures and elevate the risk of compartment syndrome in an already swollen limb. Final fluoroscopic views confirmed satisfactory alignment and stability of the humeral and radial fixations (Figures 4, 5). The total operative time was approximately 60 minutes, and there were no intraoperative complications.

Postoperatively, the limb was immobilized in an above-elbow posterior plaster slab with the elbow flexed to 90° and the forearm maintained in neutral rotation. The immediate postoperative neurovascular assessment was normal. The child was closely monitored for the development of swelling or pain out of proportion, but no signs of compartment syndrome were observed. Analgesics and limb elevation were continued for the first 48 hours, and finger movements were encouraged early.

The first follow-up at two weeks showed good wound healing and satisfactory pin sites; sutures were removed, and the slab was continued. At four weeks, early callus formation was evident radiographically. K-wires were removed at six weeks once sufficient consolidation was observed. The child was then started on gentle elbow and wrist range-of-motion exercises under supervision.

**Figure 6:**
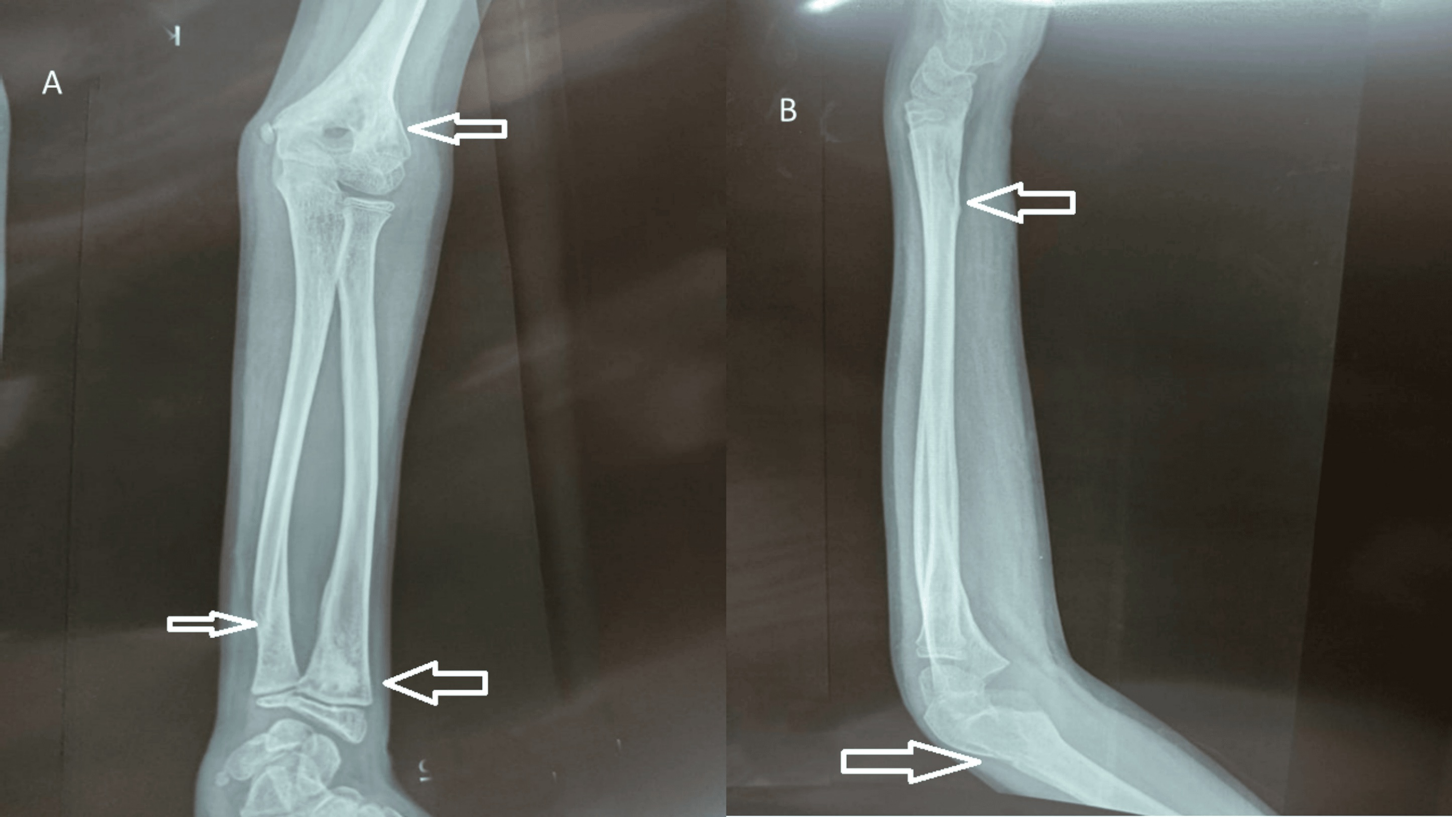
Three-month follow-up radiographs demonstrating complete fracture union and maintained alignment Anteroposterior (A) and lateral (B) radiographs of the left elbow and wrist taken at three months postoperatively showing complete bony union of the supracondylar humerus and distal radius fractures with maintained alignment. The Kirschner wires have been removed, and remodeling is evident at both fracture sites. The patient had regained full, pain-free range of motion without deformity or neurovascular deficit

**Figure 7:**
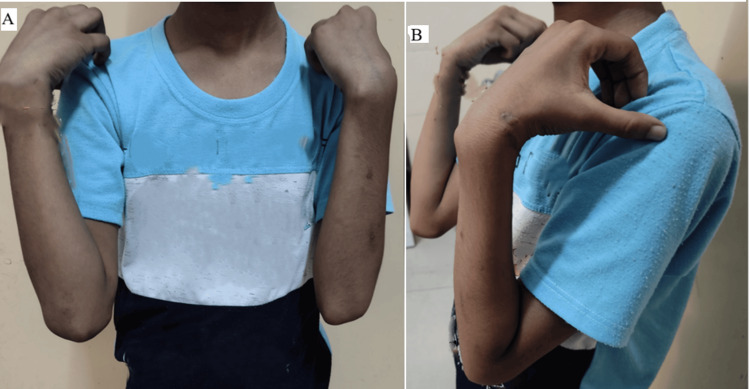
Clinical photographs demonstrating full functional recovery of the left elbow and wrist at final follow-up Clinical photographs at 12 weeks postoperatively showing the patient achieving full, pain-free flexion (A) and extension (B) of the left elbow. The range of motion is symmetrical to the opposite side, indicating excellent functional recovery and joint stability

At 12 weeks postoperatively, radiographs demonstrated complete union of both the supracondylar humerus and distal forearm fractures with satisfactory alignment (Figure 6). Clinically, at final follow-up, the patient achieved pain-free elbow motion from full extension (0°) to approximately 140°, with forearm pronation and supination of about 85° each. Wrist motion was symmetrical to the contralateral side, with approximately 75° of flexion and 65° of extension. There was no residual deformity, stiffness, or neurovascular deficit (Figure 7). At final follow-up, the carrying angle of the affected left elbow was comparable to the contralateral side and within the normal physiological range for age, with no evidence of cubitus varus or valgus deformity. However, a hypertrophic scar developed over the volar approach incision site, which remained asymptomatic and did not restrict wrist motion.

## Discussion

The pediatric floating elbow is a rare but clinically significant injury pattern that occurs predominantly following high-energy trauma. It represents a complex injury involving a supracondylar humerus fracture associated with ipsilateral forearm fractures, most commonly affecting the distal radius and ulna. This unique injury combination presents both diagnostic and management challenges for orthopedic surgeons because it involves two separate fracture sites in close proximity, often with considerable soft tissue swelling and a high potential for neurovascular compromise [1,3,6].

The mechanism of injury typically involves a fall onto an outstretched hand, which transmits an axial load up the forearm to the elbow. The resulting hyperextension force at the elbow leads to a supracondylar fracture of the humerus, while the transmitted force distally produces fractures of the radius and ulna [4]. Such injuries are most often seen in active school-aged children, with the nondominant limb more frequently involved. High-energy mechanisms such as falls from height or road traffic accidents are the usual causes [7].

Several complications were reported in association with this injury pattern, including compartment syndrome, neurovascular compromise, malunion, and joint stiffness, but recent pediatric literature suggests that floating elbow injuries are not as problematic as previously believed, with most children achieving good to excellent functional outcomes when fractures are managed appropriately and neurovascular status is preserved [5]. Compartment syndrome remains the most feared complication, with an incidence of up to 33% in some reports [6,8]. It results from increased compartmental pressure caused by combined fracture swelling or tight immobilization, potentially leading to ischemic contractures if left untreated. Neurovascular injuries, particularly to the median or anterior interosseous nerve, are not uncommon due to the displacement pattern of supracondylar fractures. Additionally, malunion, especially cubitus varus or valgus deformity, may result from improper reduction or premature removal of fixation. Elbow stiffness may also occur secondary to prolonged immobilization or soft tissue scarring.

The cornerstone of management is early and stable fixation to restore alignment and minimize complications. CRPP remains the standard approach for most supracondylar and forearm fractures in children [9]. However, the sequence of fixation remains controversial. Some authors recommend addressing the forearm fractures first to facilitate manipulation of the humerus, while others suggest fixing the humeral fracture initially to provide a stable proximal base and minimize traction on neurovascular structures during distal fixation [10,11]. In our case, the humerus was fixed first, followed by the distal radius. Selective fixation of only the more displaced forearm bone is supported by several studies, as stable, minimally displaced ulnar fractures in growing children generally remodel well without surgical intervention [5,12].

Open reduction of the distal radius was performed through a volar approach as the fracture could not be adequately reduced by closed methods due to soft tissue interposition and loss of cortical contact. Literature supports open reduction in pediatric distal radius fractures when closed reduction fails or when precise anatomical alignment is necessary to restore the normal wrist contour and prevent loss of motion [10].

Although Ditsios et al. proposed a classification for adult floating-elbow injuries based on fracture location, it is not applicable in children [12]. In pediatric populations, Templeton and Graham, and Rasool proposed descriptive classifications based on fracture site and displacement. According to these, the present case corresponds to a Templeton type II and Rasool Group II pattern, both indicating distal forearm involvement requiring surgical fixation [2,9].

Postoperative management plays an equally crucial role in ensuring favorable outcomes. Vigilant monitoring during the first 48 hours after surgery is essential to detect early signs of compartment syndrome. Elevation of the limb, maintenance of adequate padding beneath immobilization, and regular neurovascular checks are mandatory components of care. In the present case, early fixation and careful postoperative observation allowed for uneventful recovery. The patient achieved full, pain-free elbow and wrist motion by the 12th postoperative week, with excellent remodeling of the ulna. Minor postoperative complications, such as hypertrophic scarring at the surgical incision site, may occur but generally resolve without functional impairment when managed conservatively with scar massage and silicone gel application.

The overall prognosis of pediatric floating elbow injuries is generally good when managed promptly with stable fixation and close follow-up. Functional outcomes are generally excellent in pediatric floating elbow injuries when neurovascular status remains intact, and early mobilization is initiated after radiological union, as supported by pediatric series and systematic reviews [5]. Blakemore et al. support early operative stabilization of both fractures and emphasize the importance of compartment monitoring and selective fixation for optimal outcomes [4].

## Conclusions

The pediatric floating elbow is a rare and potentially serious injury that requires early recognition, careful assessment, and a well-planned surgical approach. Prompt stabilization of both fractures, either through closed or open reduction, ensures anatomical alignment, prevents secondary displacement, and minimizes the risk of neurovascular complications. In the present case, closed reduction and K-wire fixation of the supracondylar humerus fracture combined with open reduction and fixation of the distal radius, along with conservative management of a relatively stable ulnar fracture, resulted in an excellent functional outcome.

This case highlights that individualized management based on intraoperative fracture stability and soft-tissue condition can yield satisfactory results without increasing the risk of complications. Close postoperative observation for compartment syndrome and early rehabilitation remain essential components of care. Overall, timely fixation, selective stabilization, and meticulous postoperative monitoring form the cornerstones of successful treatment in pediatric floating-elbow injuries. For practicing clinicians, this case underscores that a tailored, fracture-specific approach rather than routine fixation of all components can lead to excellent functional outcomes while minimizing complications.
